# Immunocapture sample clean-up in determination of low abundant protein biomarkers – a feasibility study of peptide capture by anti-protein antibodies†

**DOI:** 10.1039/c9ra05071j

**Published:** 2019-10-29

**Authors:** Maren C. S. Levernæs, Bassem Farhat, Inger Oulie, Sazan S. Abdullah, Elisabeth Paus, Léon Reubsaet, Trine G. Halvorsen

**Affiliations:** Department Pharmacy, University of Oslo P. O. Box 1068 Blindern 0316 Oslo Norway trinegha@farmasi.uio.no +47 22854402 +47 22855735; Department of Medical Biochemistry, Norwegian Radium Hospital, Oslo University Hospital Norway

## Abstract

Immunocapture in mass spectrometry based targeted protein analysis using a bottom-up workflow is nowadays mainly performed by target protein extraction using anti-protein antibodies followed by tryptic digestion. Already available monoclonal antibodies (mAbs) which were developed against intact target proteins (anti-protein antibodies) can capture proteotypic epitope containing peptides after tryptic digestion of the sample. In the present paper considerations when developing a method for targeted protein quantitation through capture of epitope containing peptides are discussed and a method applying peptide capture by anti-protein antibodies is compared with conventional immunocapture MS. The model protein used for this purpose was progastrin releasing peptide (ProGRP), a validated low abundant biomarker for Small Cell Lung Cancer with reference values in serum in the pg mL^−1^ range. A set of mAbs which bind linear epitopes of ProGRP are available, and after a theoretical consideration, three mAbs (E146, E149 and M18) were evaluated for extraction of proteotypic epitope peptides from a complex sample. M18 was the best performing mAb for peptide capture by anti-protein antibodies, matching the LOD (54 pg mL^−1^) and LOQ (181 pg mL^−1^) of the existing conventional immunocapture LC-MS/MS method for determination of ProGRP. Peptide and protein capture using the same mAb were also compared with respect to sample clean-up, and the peptide capture workflow yielded cleaner extracts and therewith less complex chromatograms. Analysis of five patient samples demonstrated that peptide capture by anti-protein antibodies can be used for the determination of various levels of endogenously present ProGRP.

## Abstract 2

Targeted protein biomarker determination by immunocapture LC-MS/MS: comparison of peptide and protein capture using anti-protein antibodies.

## Graphical abstract

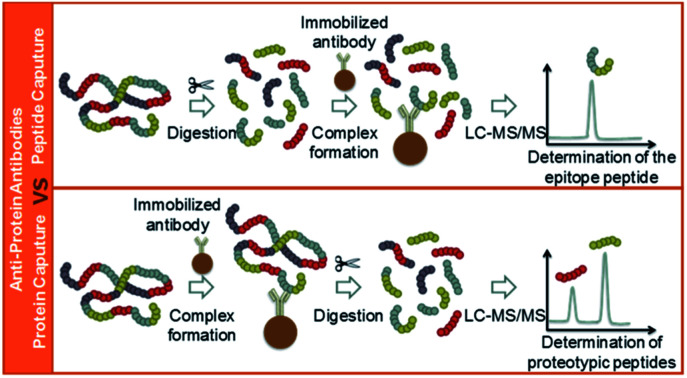

## Introduction

In recent years the proteomics society has shown a growing interest in targeted mass spectrometry (MS) based methods to quantify protein biomarkers.^1^ Despite its unique mass selectivity, sensitive detection of intact proteins by MS is limited. The protein biomarker is thus usually digested by a proteolytic enzyme to generate proteotypic peptides that are analyzed by LC-MS.^2,3^ The choice of the surrogate peptide, also known as the signature peptide or proteotypic peptide, used for the quantitation of the entire protein is crucial for the quality of the assay and should thus be unique to the targeted protein.^2–4^

Among the most promising methods to ensure high sensitivity and selectivity in targeted analysis of low-abundant protein biomarkers is a combination of immunoaffinity enrichment (immunocapture), protein digestion and LC-MS/MS detection of signature peptides.^1–6^ Immunocapture can be performed both prior to and after the protein digestion step, and provides unique selectivity through the unique interaction of the target protein or signature peptide and the capture antibody.^2^ The antibodies used for immunocapture target either linear or conformational epitopes. Linear epitopes consist of continuously neighbouring amino acid residues along the protein sequence, while conformational epitopes consist of amino acid residues that are discontinuously arranged along the protein sequence, that are brought together through folding of the polypeptide chain.^7^ Thus antibodies targeting conformational epitopes can be used for the enrichment of the intact protein, whereas antibodies targeting linear epitopes can both capture intact protein and epitope containing peptides.^5^ Additionally, anti-peptide antibodies may be produced to target signature peptides (SISCAPA).^3,5^

The greatest advantage of choosing peptide immunocapture over protein immunocapture is that peptide samples are easier to handle, due to degradation, unfolding and solubility issues often associated with full-length proteins.^5^ Quantitative, multiplexed immuno-MRM assays targeting peptides rather than proteins are expected to produce cleaner eluates and thus less interfering peptides are introduced to the LC-MS.^8^ In addition, the proteolytic step is performed prior to extraction, creating the possibility that the antibodies can be re-used and thus the cost per sample is reduced. Approaches based on peptide capture are also less likely to be influenced by auto-antibodies, as these antibodies are degraded during the proteolytic step.^9^

The use of monoclonal antibodies (mAbs), unlike polyclonal antibodies with its batch to batch variation,^8^ is especially attractive in assay development for both peptide and protein enrichment as they are renewable reagents of which the production can be standardized. The availability of validated anti-peptide mAbs for immunoaffinity enrichment of proteotypic peptides is however very limited.^10^ The number of anti-protein mAbs, on the other hand, is huge and are often commercially available due to ongoing antibody screening in pharmaceutical industry.^1^ Hence, peptide capture by anti-protein antibodies might be a promising alternative for the enrichment of proteotypic peptides without the need for time-consuming and expensive development of anti-peptide antibodies. In 2004, Zhao *et al.* characterized the epitope of an anti-troponin antibody by epitope excision and the identified (missed cleavage) peptide was used as the surrogate peptide in the targeted protein quantitation.^11^ This approach is most easily applied to antibodies with known epitopes. However, as demonstrated by Schoenherr *et al.* it can also be applied without knowledge of the epitope configuration (overall success rate 14%).^8^ The strategy was also recently applied in an on-line set-up where mAbs were covalently immobilized on acrylate-monoliths coupled to nano-LC-MS, demonstrating the possibility of automated capture and analysis of proteotypic epitope peptides.^12^ In order to successfully develop a peptide capture assay based on anti-protein antibodies, the antibody should recognize a linear epitope, not disrupted by a cleavage site. Since the majority of the commercially available antibodies used for western blot analysis and ELISA assays have linear epitopes,^13^ this approach is predicted to be widely applicable.

In the study presented here, we thoroughly explore the potential of peptide enrichment using anti-protein antibodies for LC-MS based targeted protein determination by comparing it with conventional immunocapture MS strategy. The small cell lung cancer (SCLC) biomarker progastrin releasing peptide (ProGRP) was used as model compound and immunocapture MS of intact ProGRP as benchmark. The advantage of this model system is availability of well-characterized mAbs with known epitopes that could be used for comparison. The set-up that is compared with protein extraction utilizes the same antibody as applied in the on-line study described above. However, the present study thoroughly discusses the considerations necessary for peptide extraction using protein antibodies and compares the method with methods based on immunocapture of the intact protein followed by LC-MS/MS determination of signature peptides.^14,15^ In addition, to assess the feasibility of using the proteotypic epitope peptide as the surrogate peptide in the quantitation of proteins in real samples the low abundance model biomarker ProGRP was determined in serum; both in spiked samples and samples from patients diagnosed with SCLC.

## Experimental

### Protein standards and chemicals

Cloned ProGRP isoform 1 and anti-ProGRP (mAbs E146, E149 and M18) were provided by the Central Laboratory, Norwegian Radium Hospital, Oslo University Hospital (Oslo, Norway). Trypsin (TPCK treated, from bovine pancreas, sequencing grade), Lys-C (from Lysobacter enzymogenes), and formic acid (FA) were purchased from Sigma Aldrich (St. Louis, MO, USA). All other chemicals used were of analytical grade. The stable isotopic labeled internal standard with sequence NH_2_-ALGNQQPSWDSEDSSNF(K*)–COOH (purity: >95%), where K* denotes Lys labeled with ^13^C/^15^N, was bought from Innovagen (Lund, Sweden).

### Serum samples

Human serum from healthy subjects was obtained from Oslo University Hospital, Ullevål (Oslo, Norway), and serum samples from cancer patients were supplied by the Norwegian Radium Hospital, Oslo University Hospital. All serum samples were stored at −30 °C. The use of both serum from healthy subjects and patient samples for our research purposes was performed in strict accordance to Norwegian law (“Lov om medisinsk og helsefaglig forskning (helseforskningsloven)”) and the use of patient samples was approved by the Norwegian Regional Committee for Medical Research Ethics (REK, http://helseforskning.etikkom.no). The research project is registered in the in the database for health related research at the Department of Pharmacy, University of Oslo (Oslo, Norway). Informed consent was obtained from all subjects. Methods used to analyze all serum samples were in accordance with relevant guidelines and regulations (mentioned above).

### Solutions

ProGRP isoform 1 (AA 1-125 + 8) was cloned from human cDNA (OriGene Technologies), expressed in *Escherichia coli* (Promega) using pGEX-6P-3 constructs (GE Healthcare) and purified as described elsewhere.^16^ The concentration of the ProGRP stock solution was determined by absorbance at 280 nm (A280). Working solutions were prepared by dilution with 50 mM ammonium bicarbonate solution (ABC solution) and stored at 4 °C.

Initially, complex samples were prepared by adding Lys-C or trypsin digested ProGRP to trypsin digested human serum from healthy subjects. The digested standards were added to the digested serum immediately before performing the extraction. In later experiments, spiked serum samples were prepared by adding intact ProGRP immediately before digestion with trypsin beads.

### Immobilization of trypsin to sepharose beads

Covalent immobilization of trypsin to sepharose beads was performed as described elsewhere.^17^ In brief, NHS-activated sepharose beads (NHS-activated Sepharose™ 4 Fast Flow, GE Healthcare, Uppsala, Sweden) were prewashed with 10 volumes of cold washing buffer (0.1 M phosphate buffer, pH 7.8). An equal volume of 20 mg mL^−1^ trypsin in coupling buffer (0.1 M ethanolamine, 0.25 M benzamidine and 0.1 M phosphate buffer, pH 7.8) was added and shaken (800 rpm) at room temperature for 25 min. Unbound trypsin was removed before modification buffer (0.2 M acetic acid *N*-hydroxysuccinimide (NHS)esther in 0.1 M phosphate buffer, pH 7.8) was added (1 : 1 v/v) and shaken (800 rpm) at room temperature for 20 min. The excess NHS was deactivated by blocking buffer (0.1 M ethanolamine in 0.1 M phosphate buffer, pH 8.0) (1 : 5 v/v) and shaken (800 rpm) at room temperature for 10 min. The trypsin coated beads were stored in storage buffer (50 mM Tris pH 8.2, 1 mM calcium chloride and 0.02% sodium azide) at 4 °C, protected from light. The final concentration of the immobilized trypsin beads are according to Freije *et al.*^17^ about 16.3 mg mL^−1^.

### Enzymatic digestion

In-solution digestion of ProGRP isoform 1 diluted in freshly prepared ABC solution (50 mM) was initiated by adding freshly prepared protease (trypsin or Lys-C) to give an enzyme-to-protein ratio of 1 : 40 (w/w). On-beads digestion of spiked serum samples were initiated by adding freshly prepared trypsin to give an enzyme-to-antibody ratio of 1 : 5 (w/w). Samples were incubated over night at 800 rpm at 37 °C.

Digestion of protein precipitated serum samples were initiated by adding 30 μL trypsin beads. Samples were incubated for 2 h at 800 rpm at 37 °C.

### Immobilization of monoclonal antibodies to magnetic beads

The monoclonal antibodies were covalently immobilized to tosylactivated magnetic beads (Dynabeads M280 tosylactivated, Invitrogen, Thermo Fisher Scientific, Oslo, Norway) using 1 mg of antibody to 50 mg of magnetic beads. To ensure the right orientation of the antibodies on the beads, the antibodies were added hydrochloric acid (HCl) to pH 2.5 and incubated for 1 h on ice,^18^ before neutralization by the addition of sodium hydroxide to pH 7. Coupling of the antibody to the beads was performed at pH 9.5 overnight at room temperature. The coupling volume was 1 g beads to 50 mL solution; 1/5 of final volume was 0.5 M borate buffer and added coupling buffer (50 mM trizma base, 100 mM sodium chloride, and 7.7 mM sodium azide in mqH_2_O, pH 7.5) to final volume. To remove any unbound antibody, the beads were washed twice with storage buffer (2.5 M sodium chloride, 60 mM sodium dihydrogen phosphate monohydrate, and 7.7 mM sodium azide in milliQ-H_2_O, pH 6.7) for 2 h, and once overnight, at room temperature. Antibody coated magnetic beads were stored in storage buffer (pH 6.7) at 4 °C.

### Peptide extraction

To remove any unbound anti-ProGRP the antibody-coated magnetic beads were prewashed as described elsewhere:^14^ The desired volume of beads was washed with 1 mL PBS containing 0.05% Tween 20, and re-dissolved in PBS, yielding a solution with the initial bead concentration, ready for use.

The immunoaffinity extraction was performed as follows using magnetic beads: Protein LoBind Eppendorf tubes containing the sample (an in-solution digest of the protein standard or a protein precipitated, diluted and digested serum sample) were added 20 μL of prewashed antibody-coated magnetic beads. To capture the peptides the Eppendorf tubes were rotated and shaken for 1 h on a HulaMixer (Invitrogen), to facilitate the epitope–antibody interaction. The Eppendorf tubes were then placed in the magnetic rack (DynaMag-2 from Invitrogen) to collect the beads and remove the solution. The beads were then washed with 500 μL of PBS containing 0.05% Tween 20, 500 μL of PBS, 300 μL of Tris–HCl (pH 7.4), and 300 μL of 50 mM ABC solution, prior to elution.

### Elution of extracted peptides

After immunocapture, the elution step was used for enrichment by adding 15 μL of freshly prepared 2% formic acid (FA) to the washed beads. Samples were shaken at room temperature for 5 min, placed in the magnetic rack and the supernatant containing eluted peptides was transferred to new Protein LoBind Eppendorf tubes. Additional 15 μL of 2% FA was added to the beads, shaken for 5 min and the two supernatants were collected in the same tube. The combined eluates were directly injected into the LC-MS system.

### Preparation of serum samples

Fifty μL human serum was protein precipitated using cold acetonitrile (−32 °C) in a ratio of 1 : 0.7 (v/v) with subsequent vortex mixing for 1 min and centrifugation at 10 000 rpm for 10 min. Due to its size (13 705 Da), ProGRP does not precipitate but remains in the supernatant.^19^ The supernatant was then transferred to new Protein LoBind Eppendorf tubes and diluted 1 : 40 with ABC solution (50 mM) to ensure optimal digestion conditions. Digestion was performed by adding 30 μL trypsin beads solution and the samples were incubated for 2 h at 37 °C. The samples were then centrifuged to sediment the beads and the solution was transferred to new Protein LoBind Eppendorf tubes. The internal standard was added prior to extraction. Epitope peptide extraction and elution of bound peptides were then performed as described above.

### Nano LC-MS/MS analysis

Two different nano LC-MS systems were used: an LTQ Discovery Orbitrap MS and a TSQ Quantiva, both from Thermo Fischer (Rockford, IL, US). The same columns and mobile phases were used for both systems. The samples were trapped on a C18 Acclaim PepMap 100 enrichment column (300 μm i.d. × 5 mm, 5 μm; Thermo Fischer) and further separated on a C18 Acclaim PepMap 100 analytical column (75 μm i.d. × 15 cm, 3 μm; Thermo Fischer). The loading buffer consisted of 20 mM FA : MeCN (97 : 2, v/v), and the mobile phases consisted of A: 20 mM FA : MeCN (95 : 5, v/v) and B: 20 mM FA : MeCN (5 : 95, v/v). An automatic filtration of the samples were also performed prior to trapping as described elsewhere using a steel filter (replacement screen, 1/16′′ from Teknolab, Norway).^20^

#### LTQ-Orbitrap

Twenty μL of each sample was injected into the Dionex 3000 ultimate chromatographic system. The loading mobile phase delivered the samples to the trap column with a flow rate of 10 μL min^−1^ for 4 min. The analytes were then back-flushed to the analytical column by the mobile phases with a flow rate of 0.300 μL min^−1^. Two different linear gradients were run; a short gradient from 0 to 50% B in 18 min, then increased to 100% B for 2 min before switching back to 100 A in order to regenerate the column, and a longer gradient from 0 to 50% B in 60 min, and then increased to 100% for 4 min before switching back to 100% A in order to regenerate the column. The total analysis time per run was either 31 or 89 min. The nanospray ionization source was operated in the positive ionization mode and the spray voltage was set to 2.2 kV. The heated capillary was kept at 150 °C. The capillary voltage was set at 45 V, and the tube lens offset was 100 V. Data-dependent acquisition was performed in the orbitrap mass analyser at a resolution of 30 000 over a mass range between *m*/*z* 300–2000 Da with charge state disabled. Up to six of the most intense ions per scan were fragmented by collision induced dissociation (CID) at 35% relative collision energy, activation time of 30 ms, and analysed in the linear ion trap. The fragmented *m*/*z* values were dynamically excluded for 15 s to minimize the extent of repeat sequencing of peptides and to fragment lower intensity *m*/*z* values.

#### TSQ Quantiva

Twenty μL of each sample was injected into the Dionex 3000 ultimate chromatographic system. The loading mobile phase delivered the samples to the trap column with a flow rate of 10 μL min^−1^ for 5 min. The analytes were then back-flushed from the trap column on to the analytical column with a linear gradient (starting after 5 min) from 0 to 50% mobile phase B (flow rate 0.3 μL min^−1^) in 10 min. The column was regenerated for 10 min with 100% mobile phase A before injecting the next sample. The column oven temperature was set to 60 °C to improve peak shape. Selected reaction monitoring (SRM) was performed by a TSQ Quantiva equipped with a nano-ESI source in positive mode. The spray voltage was 2250 V and the heated capillary was kept at 350 °C. Nitrogen was used as sweep gas (2 arbitrary units). The epitope peptide was fragmented at 35 V in the collision cell (argon) and selected fragments were transferred to Q3 (1005.45 → 1028.3, 1398.5).

### Data interpretation

The MS raw files were processed with Proteome Discoverer 1.4 (Thermo Fischer), using the SEQUEST algorithm, searching against a ProGRP database generated from the sequence obtained from UniProt (January, 2015). Up to five missed cleavages were considered using trypsin, Lys-C and chymotrypsin as enzymes. Methionine oxidation was chosen as variable modification. The initial parent and fragment ion maximum mass deviation was set to 10 ppm and 0.8 Da, respectively.

The Thermo Scientific Xcalibur software version 2.1 (Thermo Fischer) was used to manually extract ion chromatograms (XICs), peak area and signal intensities of selected tryptic peptides.

The extraction yield was calculated from the analysis of both the bound (eluate) and unbound (supernatant) fraction of the epitope peptide.

Evaluation of statistical significance was performed using a paired sample *t*-test, with a cut-off value of 0.05.

### *In silico* digestion and similarity search

The sequence for ProGRP isoform 1 was acquired through The National Center for Biotechnology Information (NCBI) database (accession number NP_002082.2). *In silico* digests (Protein Prospector version 5.18.1) were performed using the following parameters: trypsin or Lys-C digest, zero missed cleavages, no modifications, peptide mass 400–2000, and minimum peptide length of five amino acids. The results were used to determine which protease should be used to generate a zero missed cleavage peptide containing the intact epitope of mAb E146 and mAb E149/M18.

To investigate if the epitope containing zero missed cleavage peptides solely originated from ProGRP, an NCBI BLAST (National Center for Biotechnology Information, Basic Local Alignment Search Tool) sequence similarity search was performed and used to ensure specificity of the proteotypic epitope peptides. BLASTP 2.6.0+ was used as an algorithm with protein sequences from NCBI's Reference Sequence Project (RefSeq) as the database of choice. In addition to *Homo sapiens*, the BLAST searches were performed on *Mus musculus* and *Bos taurus*, because the antibodies and the proteases used were derived from these organisms respectively.

## Results and discussion

### Considerations in peptide capture by anti-protein antibody method development

#### *In silico* evaluation of proteotypic peptides suitable for protein antibody capture

When targeting proteotypic peptides with anti-protein mAbs, the choice of signature peptides is narrowed down to those containing the intact linear epitope. As a result, the epitope containing peptide might not be the peptide giving the highest signal intensity and best sensitivity in a digested standard sample. However, due to the efficiency and the ability for excellent clean-up of the selected mAb performing the epitope peptide extraction (as described below) the desired detection limits might still be possible to reach.

**Fig. 1:**
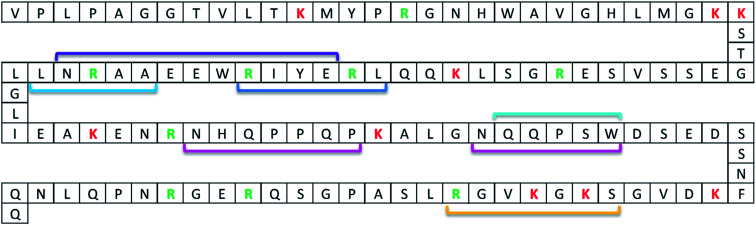
ProGRP isoform 1 sequence with binding epitopes of available anti-ProGRP mAbs. The binding epitopes have been determined by pepscan analysis. Linear epitopes: E172 (purple), E146 (dark blue), M11 and M16 (light blue), E149, M7, M8, M9, M15, M18 and M19 (turquoise), and M37 (yellow). Non-linear epitopes: E168 (pink). Trypsin cleavages sites are marked with red (lysine) and green (arginine) letters.

For the model system applied in the present paper, several anti-protein mAbs (Nordlund *et al.*^21^) which recognise and bind to different regions of our model protein ProGRP (Fig. 1) are already available. To investigate if an epitope containing zero missed cleavage peptide could be generated for the available mAbs, *in silico* digests with different enzymes (trypsin and Lys-C) were performed in Protein Prospector. This search resulted in one proteotypic epitope peptide candidate for E146, E172, M11 and M16 using Lys-C and one for E149, M7, M8, M9, M15, M18 and M19 using trypsin or Lys-C, for M37 no epitope peptide candidate was available for trypsin and Lys-C. The following BLAST query showed that the only source for the two peptides was ProGRP. Finally, SEQUEST identified the proteotypic epitope peptide candidates performed on data from FT-Orbitrap analysis on *in-solution* digests of ProGRP. Thus, the Lys-C generated peptide QQLREYIRWEEAARNLLGLIEAK and the trypsin (or Lys-C) generated peptide ALGNQQPSWDSEDSSNFK could be used as proteotypic epitope peptides for mAb E146, E172, M11 and M16 and mAbs E149, M7, M8, M9, M15, M18 and M19, respectively.

#### Choice of antibodies for further evaluation

The *in silico* screening resulted in two potential epitope peptides that could be captured using a range of possible mAbs. To narrow down the mAbs for further evaluation knowledge about equilibrium dissociation constants and mAbs used in assays was considered. Equilibrium dissociation constants were available for only a couple of the mAbs ranging from 2.63 × 10^−10^ and 1.00 × 10^−9^, reflecting very high affinity.^16^ The lowest constant (2.63 × 10^−10^) was seen for mAb E146 which also has been shown to be the best choice for capture the whole protein: mAb E146 recognizes a linear epitope covering amino acid residues 48-53 (LREYIR) and is used in combination with mAb E149, which recognize a different epitope covering amino acid residues 84-88 (QQPSW), in a very sensitive two-sided immunofluorometric assay (IFMA).^16^ The affinity of mAb E149, however, seems to be affected by conformational changes of ProGRP and other mAbs recognizing the same epitope has also been developed.^21^ Among these, mAb M18 was proven to be a good tracer outperforming the original tracer antibody, mAb E149.^22^ Based on this information mAbs E146, E149 and M18 was further assessed as potential candidates for capture of the epitope containing peptides QQLREYIRWEEAARNLLGLIEAK produced by Lys-C cleavage for capture by mAb E146 and ALGNQQPSWDSEDSSNFK produced by trypsin (or Lys-C) cleavage for capture by mAbs E149 and M18.

#### Effect of residual protease on antibody performance

One challenge related to immunocapture of proteolytic peptides is the presence of residual trypsin or Lys-C in the sample during peptide extraction. Residual proteolytic activity might compromise the performance of the antibody. This is not an issue in conventional immunocapture MS methods, as proteolysis takes place after extraction. However it has been described as a challenge for SISCAPA assays and various SISCAPA assays have solved this by adding TLCK (tosyl-l-lysine chloromethyl ketone)^23,24^ to stop the trypsin activity or by adding acid and performing an additional clean-up step (SPE, spin-filter, evaporation)^25–28^ prior to extraction.

The effect of residual enzyme on the mAbs in the present study was investigated by subjecting the antibody coated magnetic beads to various amount of proteolytic enzyme prior to extraction of intact ProGRP. The antibody coated beads E146 and M18/E149 were incubated with Lys-C (6.25 and 250 ng mL^−1^) or trypsin (6.25 ng mL^−1^, 250 ng mL^−1^ and 50 mg mL^−1^), respectively, for 2 h at room temperature. After enzyme treatment, the antibody containing beads were washed before being used in the extraction of intact ProGRP from spiked samples (250 ng mL^−1^). After extraction, on-beads trypsin digestion was carried out overnight. The quantitative yield of the proteotypic peptides generated after digestion were compared to those generated from an extraction of intact ProGRP by antibody containing beads not exposed to active enzyme prior to extraction (control).

**Fig. 2:**
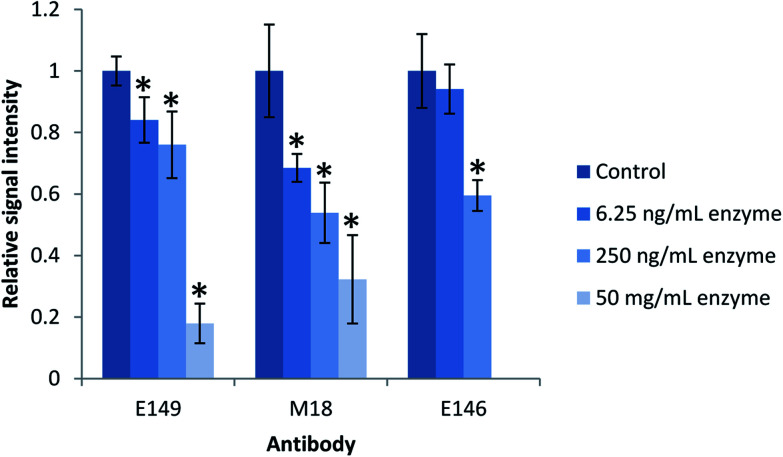
Effect of protease on antibody performance. The antibodies were incubated with active enzyme in an enzyme-to-antibody ratio of 1 : 1280 (6.25 ng), 1 : 32 (250 ng) and 6250 : 1 (50 mg) for 2 h prior to extraction of intact ProGRP (250 ng mL^−1^, *n* = 3). mAbs E149 and M18 were incubated with trypsin while mAb E146 was incubated with Lys-C. Antibody performance was evaluated by immunocapture of intact ProGRP followed by digestion on-beads overnight and subsequent LC-MS/MS analysis. Error bars represents the standard deviation. *Significantly different from the control (*P*-value < 0.05).

There was, as shown in Fig. 2, a significant decrease in the quantitative yield of ProGRP after the extraction by antibodies exposed to the proteolytic enzyme compared to the control (*P*-value < 0.05), except for the E146 beads which were more resistant towards low amounts of proteolytic enzyme (or Lys-C less efficient in digesting the antibodies) and showed no significant difference in the quantitative yield compared to the control (*P*-value > 0.05). When exposed to the amount of enzyme needed to digest a protein precipitated serum sample (50 mg mL^−1^), the antibodies still worked, but their performance (monitored by signal intensity of proteotypic peptides) was reduced to about 20–30%. The observed decrease is most likely due to digestion of the antibody. When targeting low abundant protein biomarkers, high extraction yield is needed in order to reach sufficient detection and quantitation limits. Thus, alternative digestion procedures would be beneficial to preserve the antibodies performance.

In the research presented here we circumvent the interference of residual proteolytic enzyme activity during extraction by using trypsin immobilized on beads. This allowed us to remove trypsin from the sample prior to extraction.^29^

### Proof-of-concept and evaluation of peptide capture from complex samples

A proof-of-concept study was performed for all three mAbs (E146/E149/M18) after immunocapture of an in-solution digest of ProGRP. These experiments are described in detail in the ESI Section† “Evaluation of anti-protein mAbs for peptide extraction”. Although all three antibodies were capable of extracting their proteotypic epitope peptide, M18 was superior with an extraction yield of 95%.

Subsequent to this, peptide capture was evaluated from complex samples. These experiments are described in detail in ESI Section† “Peptide extraction from complex samples”. All three antibodies were capable of extracting digested ProGRP from a complex sample (protein precipitated and digested serum). However as preparation of Lys-C immobilized beads were too expensive, and M18 outperformed E149 with respect to extraction efficiency, the following method optimization (described in detail in ESI Section† “Method optimization of peptide capture”) was performed using M18 coated magnetic beads only.

### Method evaluation

A brief evaluation of the peptide enrichment method was performed using M18 as capturing antibody and subsequent triple quadrupole mass spectrometry determination demonstrating the linearity, limit of detection (LOD) and limit of quantitation (LOQ). Serum was spiked with ProGRP concentrations ranging from 500 pg mL^−1^ to 50 ng mL^−1^ (6 concentration levels, *n* = 3), and stable isotopic labeled peptide was used as internal standard (added after digestion and prior to peptide enrichment). A linear curve (1/X) with acceptable correlation value (*R*^2^ = 0.96) was produced for the proteotypic epitope peptide (ALGNQQPSWDSEDSSNFK). The % RSD values were 13.7% or lower for all six concentration levels, using internal standard correction. This is generally considered satisfactory for bioanalytical methods.^30^ From the signal-to-noise ratio of the lowest concentration of the curve, the LOD for the ProGRP determination was estimated to be 54 pg mL^−1^ (S/N = 3), and the LOQ was estimated to be 181 pg mL^−1^ (S/N = 10). These results show the potential for a reliable detection and quantitation of the protein biomarker based on peptide capture by anti-protein antibodies.

### Comparison of peptide extraction with protein extraction

To further evaluate peptide capture by anti-protein antibodies as alternative for protein quantitation, a comparison of protein and peptide extraction with M18 was performed with emphasis on extraction efficiency, general clean-up efficiency, detection and quantitation limits.

#### Extraction efficiency

When applying anti-protein mAbs for the extraction of proteotypic epitope peptides it is of interest to investigate if the antibody is as efficient to extract the peptide as the intact protein. This was evaluated by performing the extraction of intact ProGRP (100 ng mL^−1^) from buffer (*n* = 5). After extraction the unbound fraction was removed from the beads and then both the bound (extracted) and unbound (not extracted) fraction was digested overnight and analyzed by LC-MS/MS. Similarly, an extraction of the proteotypic epitope peptide from an in-solution digest of ProGRP (100 ng mL^−1^) was performed in a buffered solution (*n* = 5). Again, both the bound and unbound fraction was analyzed by LC-MS/MS. No remains of ProGRP or epitope peptide were seen in the unbound fraction using mAb M18. The extraction yield of the intact protein was determined to 99 ± 15%, while the extraction yield of the proteotypic epitope peptide was determined to 96 ± 6%. These results indicate that there is no significant difference (*P*-value > 0.05) in the extraction efficiency between protein and peptide extraction with mAb M18. This demonstrates that by careful selection of antibody it is possible to achieve similar extraction efficiency for the proteotypic epitope peptide as for the intact protein.

#### General clean-up efficiency

As the extraction efficiency of the proteotypic epitope peptide was proven to be similar to that of intact protein extraction, peptide extraction was expected to be more beneficial as it is most likely produces cleaner extracts. To compare the general clean-up efficiency of the two methods, an extraction of both the intact protein and the proteotypic epitope peptide from ProGRP (150 ng mL^−1^) spiked serum was performed and analyzed on a LTQ-Orbitrap (*n* = 4).

**Fig. 3:**
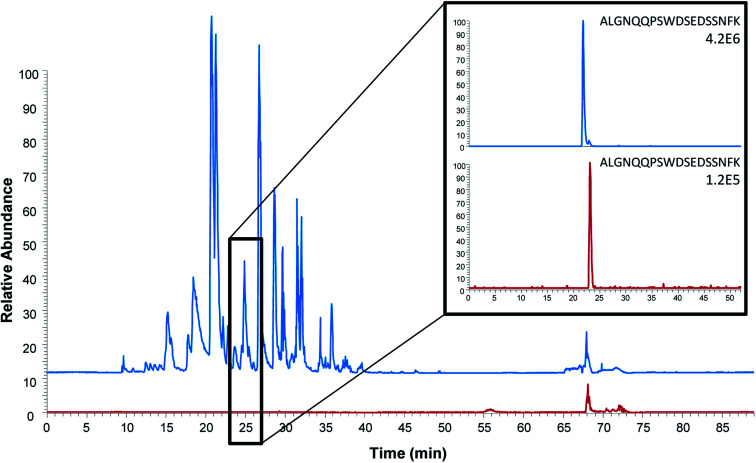
Comparison of base peak chromatograms (full scan Orbitrap analysis) after intact protein extraction (blue) and proteotypic epitope peptide extraction (red). Extracted ion chromatograms of the proteotypic epitope peptide (ALGNQQPSWDSEDSSNFK, *m*/*z* 1005.45) are shown on the right. Serum spiked with 150 ng mL^−1^ ProGRP was used as sample.

The observed background was, as expected, considerably higher after protein extraction and on-beads digestion compared to the extraction of the peptide (Fig. 3). One might expect this to be due to the digestion of the antibodies; however, only 11 out of the 111 unique peptides identified originated from the antibody M18. As also shown for on-beads digestion previously,^31^ the major contribution to the increased background was the detection of 100 unique peptides from 19 human proteins. In comparison, only three unique peptides from serum albumin were identified in addition to the epitope peptide after peptide extraction. Similar findings were done in earlier studies of protein extraction followed by elution of intact protein prior to digestion:^31^ using mAb E146 for intact protein extraction 115 unique peptides from 32 human proteins were detected after on-beads digestion while elution followed by subsequent digestion resulted in identification of 29 unique peptides from five human proteins. The observed difference may be due to the assumption that smaller proteolytic peptides to a lesser degree bind non-specific to the antibody and/or the magnetic beads compared to intact proteins. Nevertheless, these results demonstrate that peptide extraction provides the highest degree of sample clean-up. Cleaner extracts are expected to provide less matrix effects, which further will improve the sensitivity. Peptide extraction may thus be an advantage in multiplexed assays where several biomarkers are determined at once.

#### LOD and LOQ

To compare the detection and quantitation limits of peptide capture by anti-protein antibodies with that of the commonly used immunocapture of intact protein, ProGRP (5 ng mL^−1^) was extracted from serum (*n* = 5). A direct comparison was performed using mAb M18 to capture both the epitope peptide (after protein precipitation and subsequent digestion of serum) and the protein (prior to digestion). The samples were subsequently analyzed on a triple quadrupole mass spectrometer. The signal intensity of the proteotypic epitope peptide ALGNQQPSWDSEDSSNFK was considerably lower after peptide extraction compared to the signal intensity of ALGNQQPSWDSEDSSNFK after intact protein extraction. Simultaneously, the background noise in the chromatograms after peptide extraction were considerably less than in the chromatograms after protein extraction. The lower signal intensity is most probably due to the high sample complexity and thus decreased digestion efficiency, compared to the digestion of an extracted protein. While the lower background noise is most likely a result of the better sample clean-up achieved using peptide extraction (already described above and in Fig. 3). As a result the LOD (S/N = 3) and LOQ (S/N = 10), estimated using the signal intensity of ALGNQQPSWDSEDSSNFK, were lower for peptide extraction (54 pg mL^−1^ and 181 pg mL^−1^, respectively as described above) than for intact protein extraction (459 pg mL^−1^ and 1532 pg mL^−1^, respectively). Thus, the peptide capture by anti-protein antibody approach provides a detection limit below the upper reference limit of ProGRP in healthy individuals (58.9 pg mL^−1^),^22^ whereas protein extraction and detection of the same peptide (ALGNQQPSWDSEDSSNFK) with the same antibody (M18) does not.

Another method for determination of ProGRP in serum using immunocapture of intact protein, digestion and LC-MS/MS has previously been described.^14^ This method is also capable of determining ProGRP at its reference level in serum (estimated LOD and LOQ of 48 pg mL^−1^ and 162 pg mL^−1^). The obtained LOD and LOQ of the peptide capture method is hence comparable to this while the LOD and LOQ of protein capture performed as described above is not (approximately 10-fold higher LOD and LOQ). However, the methods are not directly comparable due to several differences. First, the antibody used for protein capture differs, mAb E146 is used in the previously published method while mAb M18 is used in the work described above. Secondly, two different signature peptides are monitored, NLLGLIEAK in the previously published method *vs.* ALGNQQPSWDSEDSSNFK in the present method, and it is previously seen that the signal intensity of NLLGLIEAK is higher than for ALGNQQPSWDSEDSSNFK after a digest (*i.e.* better ionization or better digestion outcome).^12^ Thirdly, the sample volume differs, 1000 μL in previously published method *vs.* 50 μL in the present method. Finally, two different LC-MS/MS systems are used the previously published methods employs an older triple quadrupole (TSQ Quantum Access, Thermo Scientific) coupled to a micro-LC while in the present paper a newer triple quadrupole (TSQ Quantiva) coupled to a nano-LC is used.

### Analysis of patient samples

**Fig. 4:**
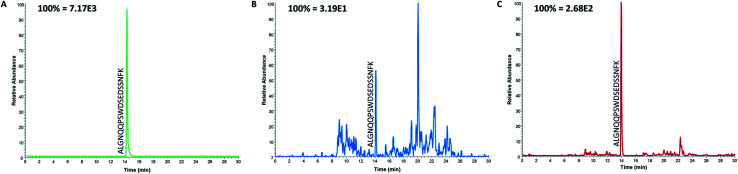
Extracted ion chromatograms of the proteotypic epitope peptide after the analysis of serum samples from a spiked standard containing 100 ng mL^−1^ ProGRP (A), a healthy individual (B) and from a patient with SCLC (C).

Even though the purpose of this study was to evaluate the applicability of anti-protein mAbs for immunocapture of proteotypic epitope peptides and not to develop a new assay for the determination of ProGRP, it was important to demonstrate the applicability of peptide extraction in real samples. Serum samples from both healthy individuals and five patients diagnosed with SCLC (in the range 0.45 ng mL^−1^ to 2.6 ng mL^−1^) were thus analyzed using the optimized workflow. The proteotypic epitope peptide was detected in serum from both healthy individuals (Fig. 4B) and patients suffering from SCLC (Fig. 4C), demonstrating that the approach can be used for detection of a wide range of ProGRP levels. Compared to the routinely used immunoassays (TR-IFMA), the ProGRP levels estimated using the peptide capture and LC-MS/MS method was approximately 10 times higher. There may be several reasons for this difference; however, due to the small number of samples no real method comparison can be performed. Nevertheless, these results demonstrated that peptide capture by anti-protein antibodies was successful also in real samples from patients suffering from SCLC.

## Conclusions

In this study we have demonstrated that peptide capture of proteotypic epitope peptides by anti-protein antibodies after protein digestion of target protein is a promising workflow for targeted biomarker determination.

Compared to a previously validated LC-MS/MS method for the immunocapture of intact protein the results demonstrated that peptide extraction provided cleaner extracts, similar sensitivity and hence most likely less matrix effects compared to the extraction of intact protein. These qualities will make peptide extraction an attractive method for multiplexed assays where several biomarkers are determined at once. The developed peptide capture method targeting the proteotypic epitope peptide of the SCLC marker ProGRP was successfully applied to the analysis of the five patient samples, and proved that the assay was able to detect different levels of endogenous ProGRP.

Based on these results, it is expected that the approach successfully can be applied to other protein biomarkers. The huge variety of and easy access to commercially available anti-protein antibodies allows for a wide application area. Peptide extraction using monoclonal anti-protein antibodies targeting linear epitopes has great potential in targeted proteomic approaches as it might facilitate the enrichment of proteotypic (or signature) peptides without the need for time-consuming and expensive development of anti-peptide antibodies, independently from batch-to-batch variations.

## Data availability statement

The data sets generated during and/or analysed during the current study are available from the corresponding author on reasonable request.

## Author contributions

The manuscript was written through contributions of all authors. All authors (Maren C. S. Levernæs, Bassem Farhat, Inger Oulie, Sazan S. Abdullah, Elisabeth Paus, Léon Reubsaet, and Trine G. Halvorsen) have given approval to the final version of the manuscript.

## Conflicts of interest

The authors declare that there are no conflicts to declare.

## Supplementary Material

RA-009-C9RA05071J-s001
