# Supplementary material for: Immunocapture sample clean-up in determination of low abundant protein biomarkers – a feasibility study of peptide capture by anti-protein antibodies
Source: RSC Adv. 2019 Oct 29;9(60):34902–11. doi: 10.1039/c9ra05071j (PMC9097496; doi:10.1039/c9ra05071j)
Supplement: RA-009-C9RA05071J-s001 [file RA-009-C9RA05071J-s001.pdf]

Electronic Supplementary Material (ESI) for RSC Advances.
This journal is © The Royal Society of Chemistry 2019

Supporting information

Immunocapture sample clean-up in determination of low abundant protein biomarkers – A feasibility study of peptide capture by anti-protein antibodies

Maren C. S. Levernæs[1], Bassem Farhat[1], Inger Oulie[1], Sazan S. Abdullah[1], Elisabeth Paus[2], Léon Reubsaet[1], Trine G. Halvorsen[1]*

[1]Department of Pharmacy, University of Oslo, Norway

[2]Department of Medical Biochemistry, Norwegian Radium Hospital, Oslo University Hospital, Norway

Contents

Evaluation of anti-protein mAbs for peptide extraction 2

Peptide extraction from complex samples 3

Method optimization of peptide capture 5

Digestion 5

Protein precipitation 6

References 6

Evaluation of anti-protein mAbs for peptide extraction

To demonstrate that the antibodies were capable of extracting proteotypic epitope peptides, immunocapture of an *in-solution* digest of ProGRP was performed, using Lys-C for E146 and trypsin for E149 and M18. Figure S1 shows all peptides generated in the digestion (above sequence) and those peptides being extracted using the different anti-protein antibodies (below sequence). It is evident that all three anti-protein mAbs could be used for proteotypic epitope peptide extraction (as they selectively extracted peptides containing the epitope from a digested sample). The epitope peptide of E146 is rather long (23aa; QQLREYIRWEEAARNLLGLIEAK, figure S1A) and dependant on Lys-C for generation. A high concentration (2.5 µg/mL) was needed to obtain a sufficiently high MS signal after in solution digestion. This, in addition to an extraction yield of 31 %, would make it challenging to make a sensitive enough assay based on peptide extraction with E146. The proteotypic epitope peptide of E149 and M18 on the other hand, produced a satisfactory MS signal at a lower concentration (250 ng/mL) after in solution digestion compared to the proteotypic epitope peptide of E146. The extraction yield of E149 was however considerably lower than the extraction yield of M18, as also shown by Nordlund et al[20], with 15 % and 95 % respectively. The observed difference may be due to conformational changes upon digestion that may affect the antibody's affinity towards the epitope (E149 has been demonstrated to be affected by conformational changes of ProGRP as described in the main body of the paper). This demonstrates the importance of conformation of the target molecule for efficient extraction. In addition to the zero missed cleavage peptide, two missed cleavage peptides were extracted with M18 and E149 (figure S1B and S1C). Incomplete digestion and varying production of a peptide can be a challenge when used as a signature peptide for quantitation of the intact protein. The production of the zero missed cleavage peptide and the missed cleavage peptides containing the epitope of M18/E149 was however reproducible (4.7 % RSD, n =5). These results showed the potential for a successful development of a peptide capture assay based on anti-protein antibodies, with ProGRP and mAb M18 as model system.

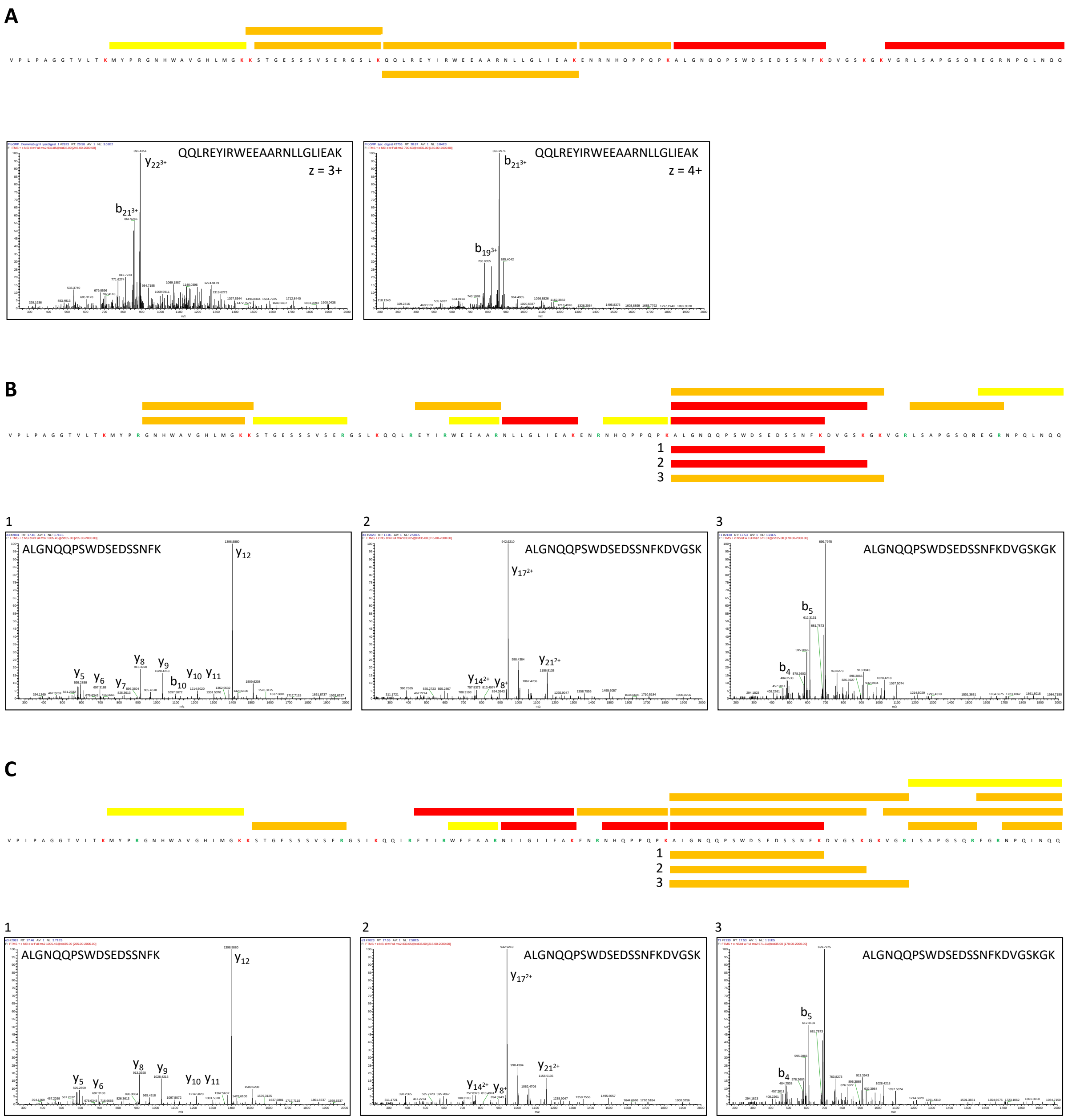

Figure S1. Performance of E146 (A), M18 (B) and E149 (C) in epitope peptide extraction. Peptides generated and detected from an in solution digest of ProGRP (Lys-C digest in A and trypsin digests in B and C) are displayed above each sequence and peptides detected after elution from the antibody after peptide extraction of the same digest are displayed below each sequence. Lys-C cleavages sites are marked with red (lysine) and trypsin cleavages sites are marked with red (lysine) and green (argenine) letters. Zero missed cleavages peptides are closest to the sequence. Signal intensity: low (yellow), medium (orange) and high (red). The MS/MS spectra confirm the identity of the peptides identified in the eluate.

Peptide extraction from complex samples

Even though the selective extraction of proteotypic epitope peptides from an in-solution digest in buffer was promising, extraction from more complex samples was needed to evaluate its performance.

Initially, the possibility of digesting ProGRP spiked serum samples directly using trypsin beads, without any kind of depletion, was briefly investigated using mAb M18. This resulted in a clean but relatively low signal for the proteotypic epitope peptide ALGNQQPSWDSEDSSNFK thus showing that this peptide could be extracted in this way from complex samples too. It was assumed that this relative low signal was due to inefficient tryptic digestion. To improve this, a previously[28-29] successfully protein precipitation was included in the workflow for initial sample clean-up. ProGRP is a small protein that will remain in solution after precipitation with the right amount of acetonitrile. Thus the final sample preparation involved protein precipitation, digestion (with enzyme immobilized on beads), peptide extraction and elution. When targeting bigger proteins, this approach may however not be feasible and digestion protocols like the recently described 'addition only' by Razavi et al[21] should be considered.

Based on these results, three different experiments were performed, primarily to confirm that selective extraction of the proteotypic epitope peptide was possible from complex samples, but also to make a survey of potential loss of analyte. First, peptide extraction of Lys-C or trypsin digested ProGRP added to protein precipitated and trypsin digested serum was performed. This gives an impression on the performance of peptide extraction from complex samples after protein precipitation. Second, peptide extraction from a sample where intact ProGRP was added to protein precipitated serum prior to tryptic digestion was performed. This would show that ProGRP could be digested (with trypsin) and extracted from a more complex sample. Finally, representing the final and complete sample preparation, intact ProGRP was added to serum prior to protein precipitation, followed by tryptic digestion and peptide extraction. For mAb E146, only the first experiment was performed as it was too expensive to make beads immobilized with Lys-C.

The results showed that it was possible to extract the proteotypic epitope peptides from a complex sample using all three anti-protein mAbs (Figure S2). A decrease in the signal intensity of the proteotypic epitope peptide was however observed after extraction with both E149 and M18 with increased sample preparation complexity. This indicated a loss of analyte both during protein precipitation and digestion. For M18, the major contributor to the loss of signal intensity was the digestion, with a recovery of 52 % (14 % RSD), while protein precipitation was the major contributor to the between sample variation, with 25 % RSD (54 % recovery). The same trend was observed for E149. The loss of signal intensity due to incomplete digestion was higher than for M18, with a recovery of 38 % (16 % RSD). In addition, the protein precipitation contributed to both an additional loss of signal intensity (18 % recovery) and increased between sample variations, with 28 % RSD. These results indicated that both the digestion and protein precipitation step could be optimized in order to increase the digestion efficiency and reduce between sample variations.

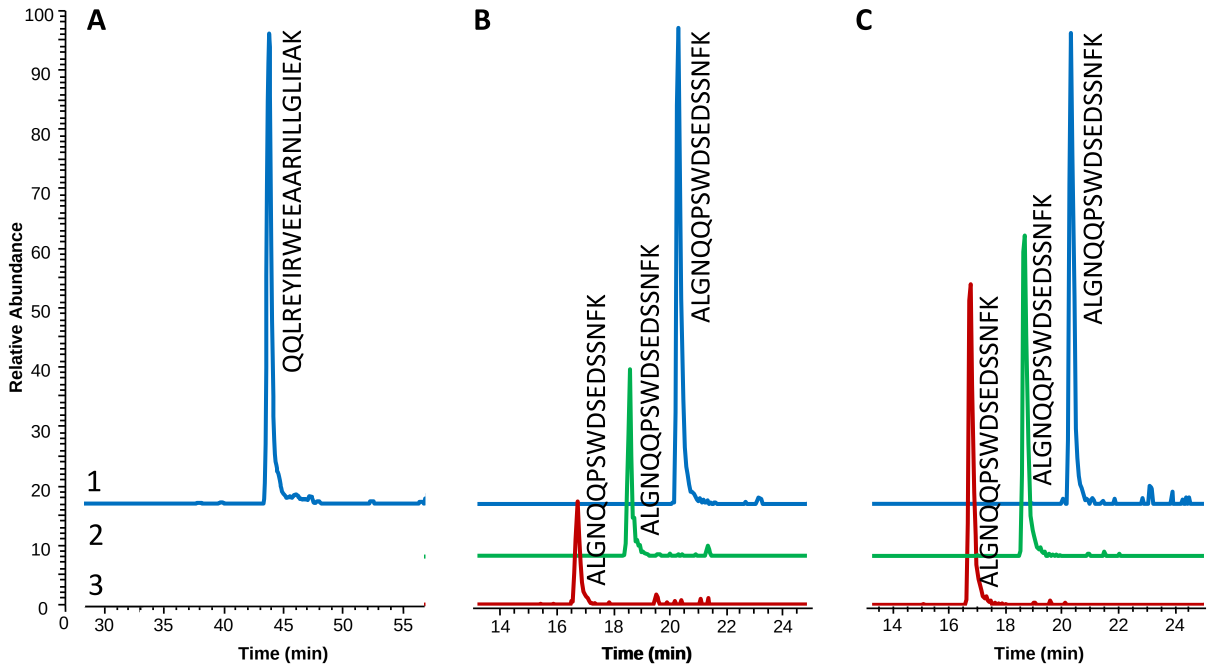

Figure S2. Relative abundance of the proteotypic epitope peptide from complex samples were 1) digested standard was added to digested protein precipitated serum, 2) intact standard was added to protein precipitated serum prior to digestion and 3) intact standard was added to serum prior to protein precipitation and digestion, with A) mAb E146, B) mAb E149 and C) mAb M18 respectively. For mAb E146, only the first experiment was performed. Offset is used on both x-axes and y-axes for better visualization of the results.

Method optimization of peptide capture

In order to detect the low levels of endogenous ProGRP in serum, optimization of both digestion (with trypsin beads) and protein precipitation were performed. This optimization was done only with mAb M18 as it outperformed mAb E149 based on extraction yield and thus signal intensity of the proteotypic epitope peptide.

Digestion

Initial experiments with immobilized trypsin were performed using 10 µL beads per sample. In order to ensure efficient digestion, optimization was performed by comparing different amount of beads (10 µL, 15 µL and 30 µL) and different incubation times (2 h, 4 h, and overnight). The highest signal intensity of the proteotypic epitope peptide was observed after digestion with 30 µL beads. There was however no apparent difference between the three time points and to speed up the sample preparation a 2 h digestion was thus chosen (data not shown).

Protein precipitation

There are several methods that are commonly used to reduce the complexity of a serum sample prior to digestion. The most used serum depletion methods in SISCAPA and related assays are depletion by filters[1-2], columns[3-4] and protein precipitation[5-6]. Protein precipitation has previously been used in the sample clean-up of ProGRP[6-7]. Protein precipitation is a procedure that involves two steps that may contribute to variation and loss of analyte; precipitation and evaporation. Winther et al[7] and Rossetti et al[6] have previously demonstrated that the protein precipitation step can be a challenge and cause of variation in the determination of ProGRP. In order to reduce the between sample variation originating from the protein precipitation step the following was investigated; 1) digestion directly in the diluted supernatant without the evaporation step and 2) optimization of the amount of acetonitrile used for precipitation.

There is some documentation that indicates that trypsin is unaffected by the presence of acetonitrile[8-9], and dilution of the supernatant (1:20 and 1:40) after protein precipitation was thus investigated as it would eliminate potential analyte loss during to the evaporation step/reconstitution step. Dilution of the supernatant with ABC buffer in a ratio of 1:40 provided the highest signal intensity and the lowest RSD values (data not shown).

To ensure that the majority of ProGRP remained in the solution after protein precipitation, an optimization of the amount of acetonitrile used was performed (data not shown). The signal intensity of the proteotypic epitope peptide reached its maximum when serum was precipitated with a ratio of 1:0.7. This ratio is slightly lower than used for ProGRP isoform 1 previously (1:0.75)[10]. The observed difference may be due to the following dilution of the acetonitrile fraction with ABC buffer (1:40) prior to digestion in this work. These common efforts increased the signal intensity and reduced the between sample variation from 30-50 % RSD to 10 % RSD (data not shown).

References

1. Anderson, N. L.; Anderson, N. G.; Haines, L. R.; Hardie, D. B.; Olafson, R. W.; Pearson, T. W., *J. Proteome Res.* **2004**, *3*, 235-244.
2. Whiteaker, J. R.; Zhao, L.; Zhang, H. Y.; Feng, L.-C.; Piening, B. D.; Anderson, L.; Paulovich, A. G., *Anal. Biochem.* **2007**, *362*, 44-54.
3. Xu, Q.; Zhu, M.; Yang, T.; Xu, F.; Liu, Y.; Chen, Y., *Clin. Chim. Acta* **2015**, *448*, 118-123.
4. Ahn, Y. H.; Lee, J. Y.; Lee, J. Y.; Kim, Y.-S.; Ko, J. H.; Yoo, J. S., *J. Proteome Res.* **2009**, *8*, 4216-4224.
5. Kushnir, M. M.; Rockwood, A. L.; Roberts, W. L.; Abraham, D.; Hoofnagle, A. N.; Meikle, A. W., *Clin. Chem.* **2013**, *59*, 982-990.
6. Rossetti, C.; Abdel Qader, A.; Halvorsen, T. G.; Sellergren, B. ; Reubsaet, L., *Anal. Chem.* **2014**, *86*, 12291-12298.
7. Winther, B.; Moi, P.; Paus, E.; Reubsaet, J. L. E., *J. Sep. Sci.* **2007**, *30*, 2638-2646.
8. Strader, M. B.; Tabb, D. L.; Hervey, W. J.; Pan, C.; Hurst, G. B., *Anal. Chem.* **2006**, *78*, 125-134.

9. Freije, J. R.; Mulder, P. P.; Werkman, W.; Rieux, L.; Niederlander, H. A.; Verpoorte, E.; Bischoff, R., *J. Proteome Res.* **2005**, *4*, 1805-1813.
10. Rossetti, C.; Świtnicka-Plak, M. A.; Halvorsen, T. G.; Cormack, P. A.; Sellergren, B.; Reubsaet, L., *Sci. Rep.* **2017**, *7*, 44298.
